# Left ventricular outflow tract stenosis following Bentall procedure using the graft insertion technique: a case report

**DOI:** 10.1186/s44215-026-00265-2

**Published:** 2026-06-23

**Authors:** Koki Yokawa, Kazunori Yoshida, Ko Ishimoto, Taku Nakagawa, Tomonori Higuma, Yosuke Tanaka, Yoshihiro Oshima, Hidefumi Obo, Hidetaka Wakiyama

**Affiliations:** Department of Cardiovascular Surgery, Kakogawa Central City Hospital, 439, Kakogawa-cho Honmachi, Kakogawa City, Hyogo 675-8611 Japan

**Keywords:** Graft insertion technique, Aortic root pseudoaneurysm, Redo aortic root surgery, Left ventricular outflow tract stenosis, 4D-flow magnetic resonance imaging

## Abstract

**Background:**

The graft insertion technique is an effective choice for redo aortic root reconstruction in cases with extensive annular destruction. Nevertheless, its impact on postoperative left ventricular outflow tract (LVOT) geometry and flow dynamics has not been investigated in detail.

**Case presentation:**

A 69-year-old woman who underwent aortic valve replacement for infective endocarditis developed prosthetic valve dehiscence with paravalvular leakage and a large aortic root pseudoaneurysm after 4 months. Because the native annulus was completely destroyed, redo aortic root replacement using the Bentall procedure with the graft insertion technique was performed. Intraoperative examination revealed no macroscopic evidence of active infection but raised concerns regarding potential LVOT narrowing after the insertion of the inverted graft. Postoperative contrast-enhanced computed tomography demonstrated significant LVOT narrowing, with a minimal diameter of 14 mm, despite the implantation of a 19-mm bioprosthetic valve. Moreover, four-dimensional flow (4D-flow) magnetic resonance imaging (MRI) revealed accelerated systolic flow at the narrowed LVOT, indicating functional LVOT stenosis.

**Conclusions:**

This case emphasizes an important pitfall of the graft insertion technique—postoperative LVOT narrowing caused by the intraventricular portion of the inverted graft. Postoperative morphological and hemodynamic evaluation using advanced imaging modalities, including 4D-flow MRI, may be required to ensure the safety of this technique in complex aortic root reconstruction.

## Introduction

Redo aortic root surgery after infective endocarditis complicated by annular destruction remains technically challenging. Extensive debridement is mandatory to eradicate infection; however, once the annular continuity is lost, secure proximal reconstruction becomes difficult, and conventional techniques may fail. The graft insertion technique has been introduced as a salvage approach to achieve secure proximal fixation in such complex situations. We report a case of redo Bentall surgery using the graft insertion technique, in which postoperative left ventricular outflow tract (LVOT) stenosis was demonstrated by multimodality imaging.

## Case presentation

**Fig. 1:**
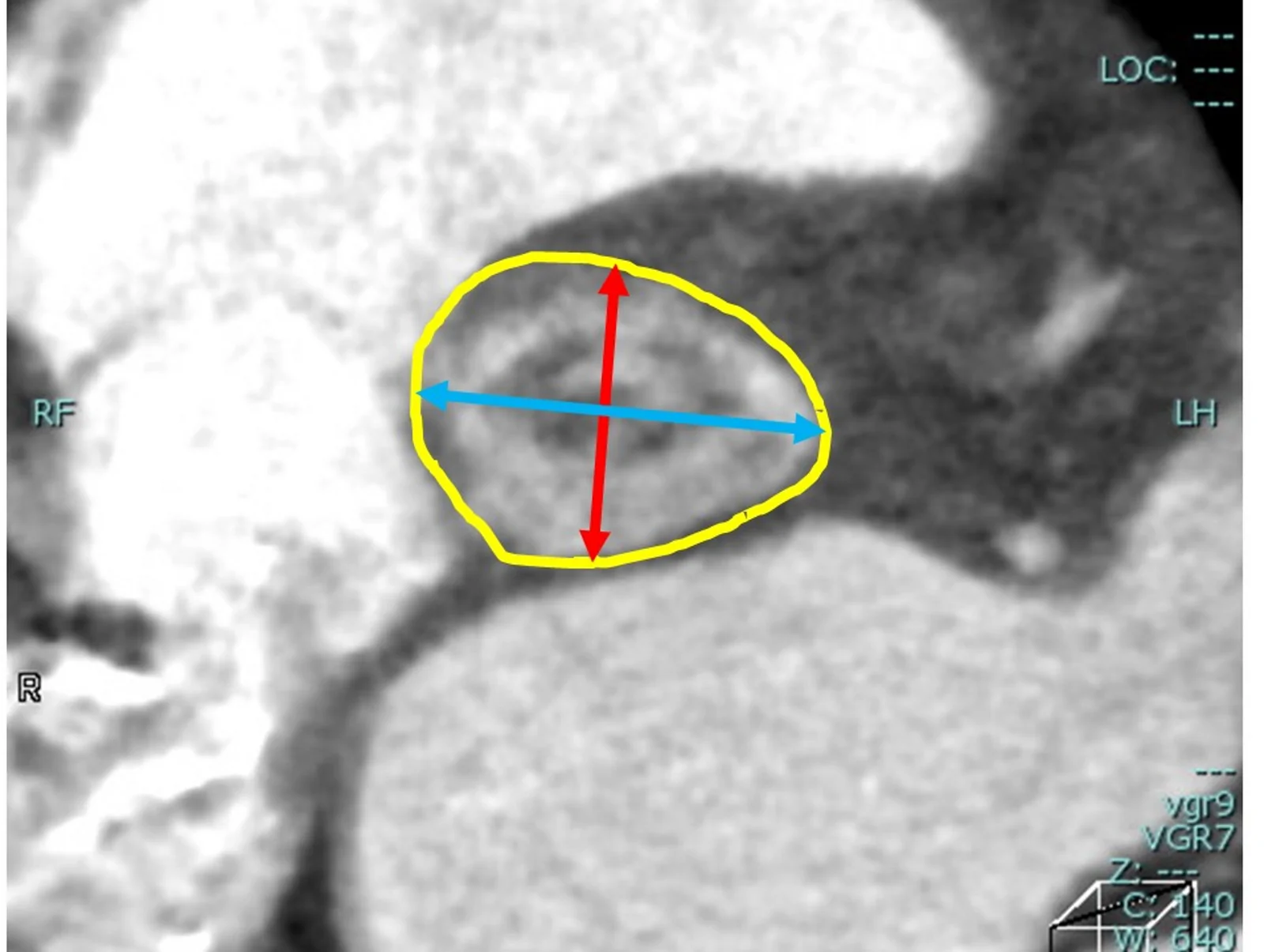
Preoperative computed tomography before the initial aortic valve replacement. Measurement of the left ventricular outflow tract (LVOT) before the initial surgery for infective endocarditis. The LVOT is outlined by the yellow line. The maximum diameter is indicated by the red line, and the minimum diameter is indicated by the blue line. The LVOT circumference was 83.2 mm, with a maximum diameter of 30.9 mm and a minimum diameter of 21.7 mm, corresponding to an area of 5.18 cm^2^. A low-attenuation lesion within the LVOT is suggestive of vegetation

A 69-year-old woman had undergone aortic valve replacement 4 months earlier for infective endocarditis. A 21-mm bioprosthetic valve (INSPIRIS; Edwards Lifesciences) had been used at the initial aortic valve replacement. Preoperative contrast-enhanced computed tomography performed prior to the initial surgery demonstrated the baseline left ventricular outflow tract (LVOT) geometry, with a circumference of 83.2 mm, a maximum diameter of 30.9 mm, and a minimum diameter of 21.7 mm, corresponding to an area of 5.18 cm^2^ (Fig. 1).

**Fig. 2:**
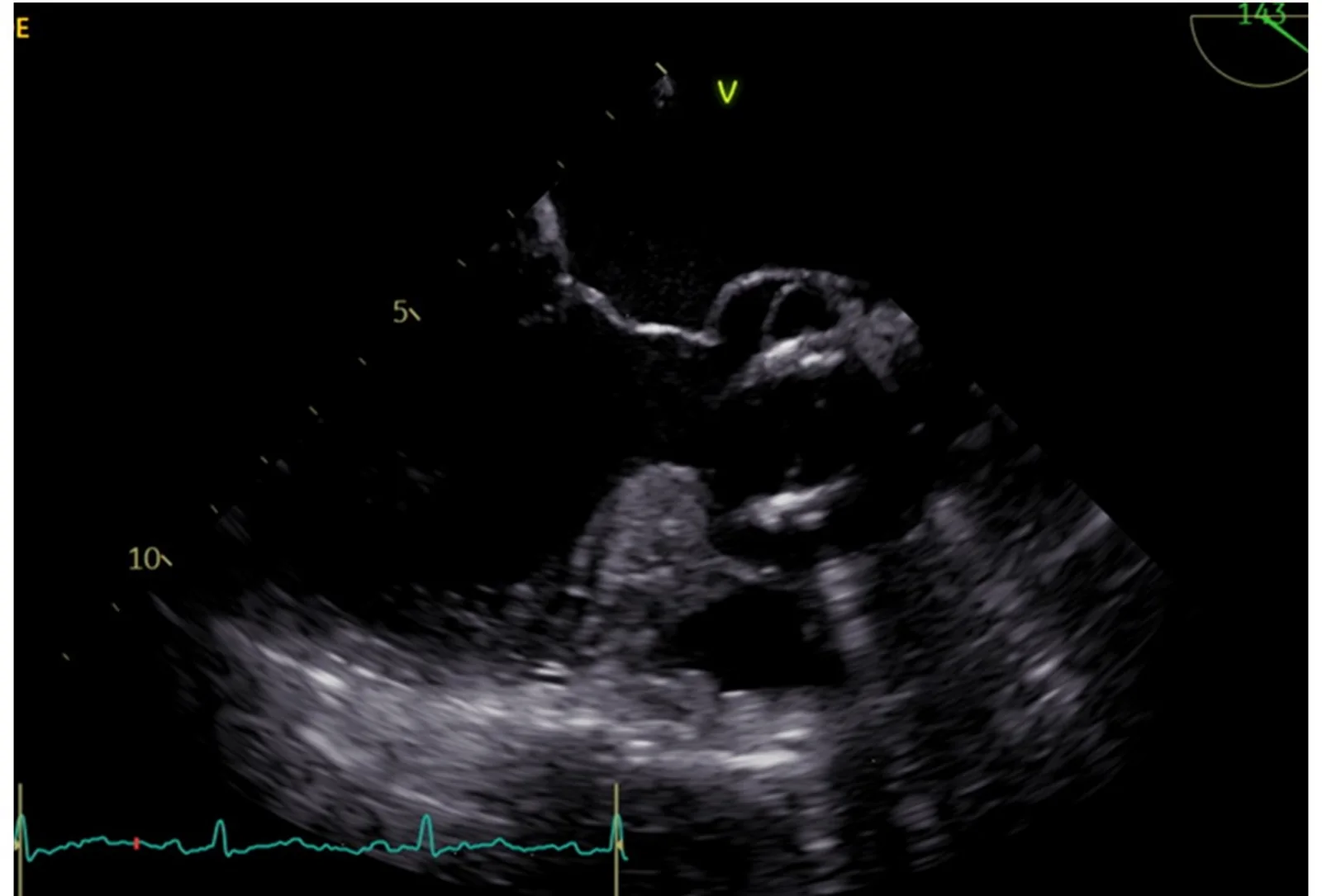
Preoperative transesophageal echocardiography. Preoperative transesophageal echocardiography showing marked rocking motion of the prosthetic aortic valve, suggesting prosthetic valve dehiscence and annular disruption

**Fig. 3:**
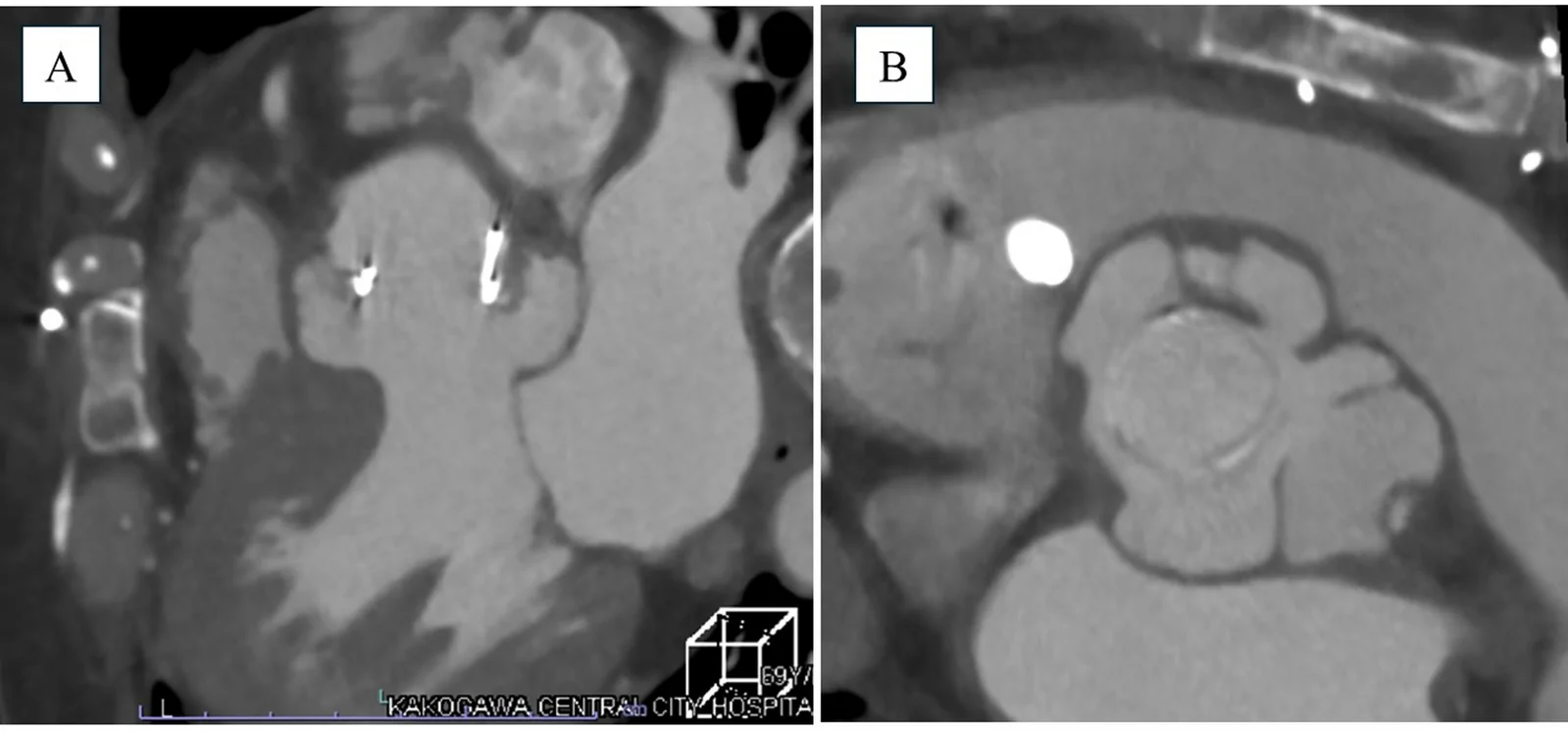
Preoperative contrast-enhanced computed tomography. **A** Long-axis view of the aortic root showing pseudoaneurysm formation at the annular level of the left and right coronary cusps. **B** Short-axis view perpendicular to the aortic annulus demonstrating pseudoaneurysm cavities surrounding the detached prosthetic valve

During follow-up, abnormal mobility of the prosthetic aortic valve was initially detected on transthoracic echocardiography. Transesophageal echocardiography was performed for further evaluation, which demonstrated marked rocking motion of the prosthetic valve, suggesting prosthetic valve dehiscence (Fig. 2). Subsequently, contrast-enhanced computed tomography revealed pseudoaneurysm formation at the annular regions of the left and right coronary cusps (LCC and RCC, respectively), with detachment of the prosthetic valve from the annulus (Fig. 3A, B). Surgical reintervention was indicated due to the risk of rupture and progressive prosthetic valve instability.

**Fig. 4:**
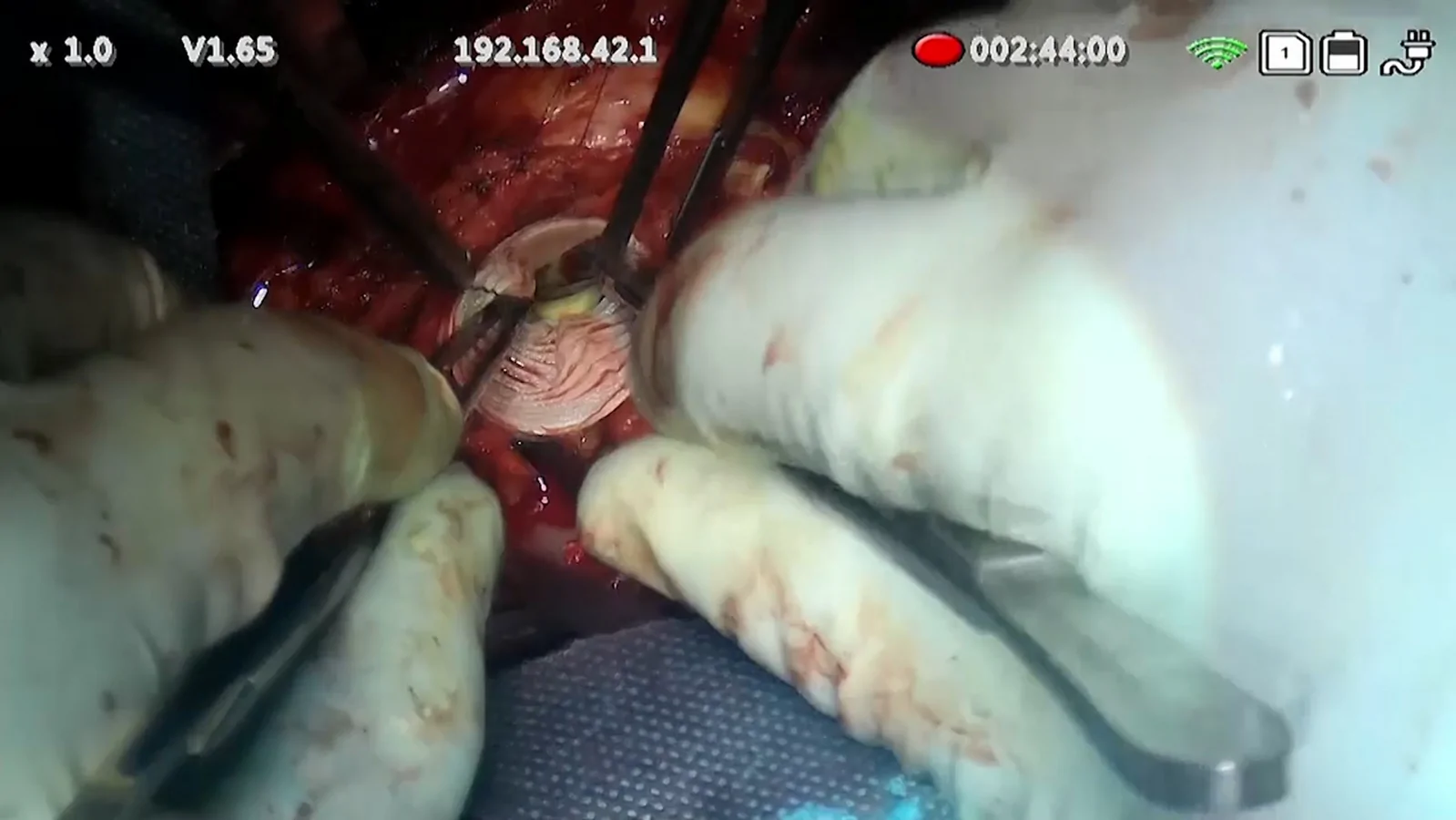
Intraoperative findings during aortic root reconstruction. Intraoperative photograph obtained during aortic root reconstruction using the graft insertion technique. The inverted vascular graft is inserted into the left ventricular outflow tract (LVOT) for proximal fixation. At this stage, narrowing of the LVOT was visually suspected due to the intraventricular portion of the graft and reinforcement materials

Redo median sternotomy was attempted; however, a major right ventricular injury occurred during reentry. Emergent cardiopulmonary bypass was established, and the chest was safely reopened under circulatory support. Intraoperative examination revealed complete loss of the aortic wall at the LCC and RCC annular regions corresponding to the pseudoaneurysm cavities, but no macroscopic evidence of active infection was found. Because the annulus was extensively destroyed, aortic root reconstruction using the graft insertion technique was selected. Intraoperative assessment showed that a 19-mm sizer could pass through the annular region, whereas a 21-mm sizer could not, suggesting an effective annular diameter between 19 mm and 21 mm. During insertion of the inverted vascular graft into the LVOT, narrowing of the LVOT was visually suspected intraoperatively (Fig. 4). A donut-shaped felt strip and bovine pericardial patch were placed for epicardial reinforcement. A vascular graft (24-mm Valsalva graft; Gelweave, Terumo Aortic) was inverted using the straight tubular portion of the graft, not the sinus segment, and inserted into the left ventricular cavity, and 11 pledgeted 2 − 0 braided polyester sutures were placed circumferentially from the left ventricular cavity toward the epicardial side and tied securely. A ball sizer or Hegar dilator was not used during knot tying. The proximal anastomosis was further reinforced with a continuous 4 − 0 polypropylene suture. The graft was then pulled out and anastomosed to a composite graft with a 19-mm bioprosthetic valve (INSPIRIS; Edwards Lifesciences). BioGlue was not used at the proximal anastomosis. During the procedure, injury to the right coronary artery required additional coronary artery bypass grafting using a saphenous vein graft, and the injured right ventricular wall was repaired with a bovine pericardial patch. The total operation time was 423 min, the cardiopulmonary bypass time was 315 min, and the aortic cross-clamp time was 189 min.

**Fig. 5:**
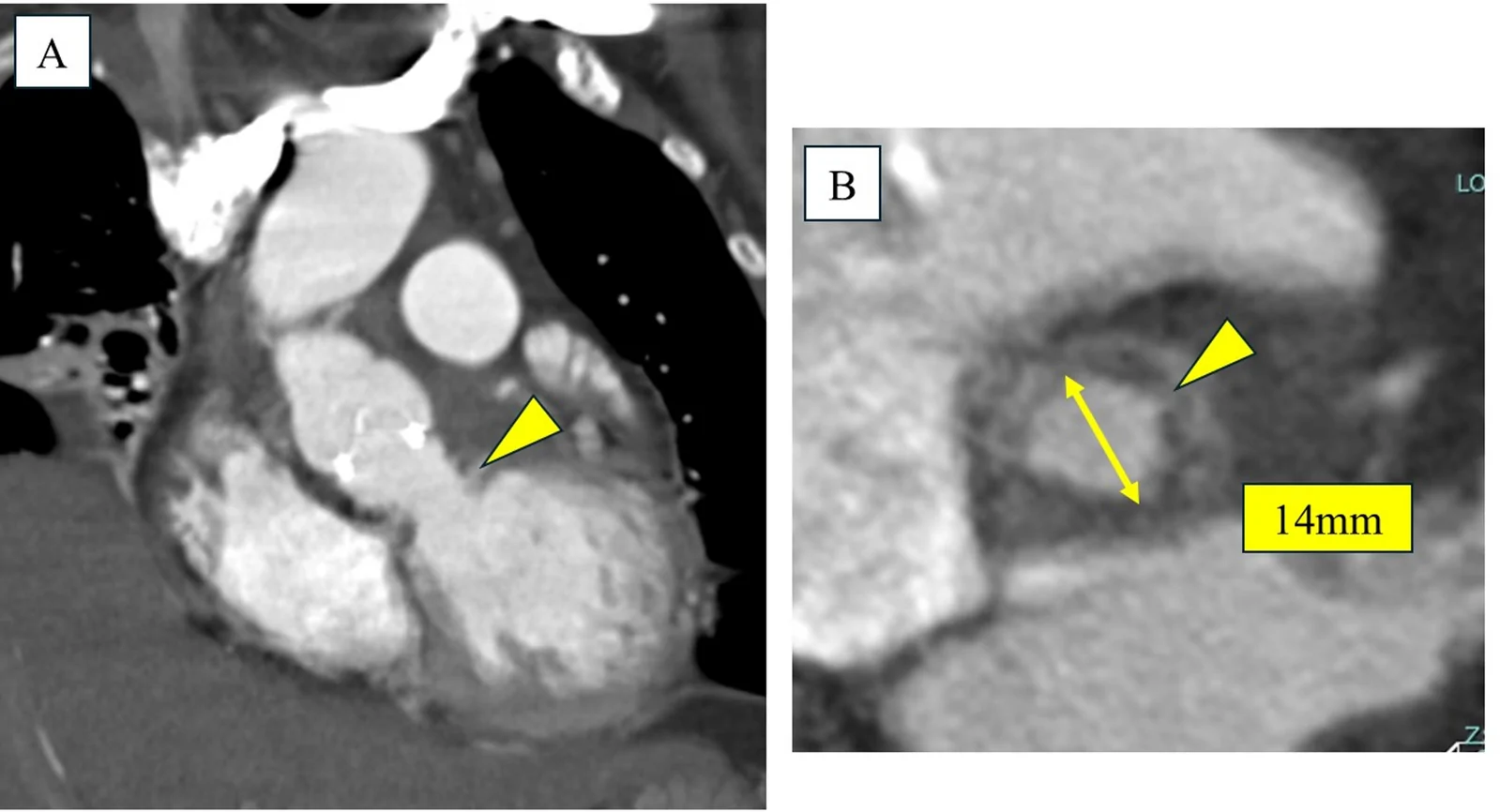
Postoperative contrast-enhanced computed tomography. **A** Long-axis view of the reconstructed aortic root after redo Bentall surgery using the graft insertion technique. **B** Short-axis view perpendicular to the left ventricular outflow tract. The yellow arrowhead indicates significant narrowing of the LVOT at the level of the inverted graft. The minimal LVOT diameter was measured as 14 mm

**Fig. 6:**
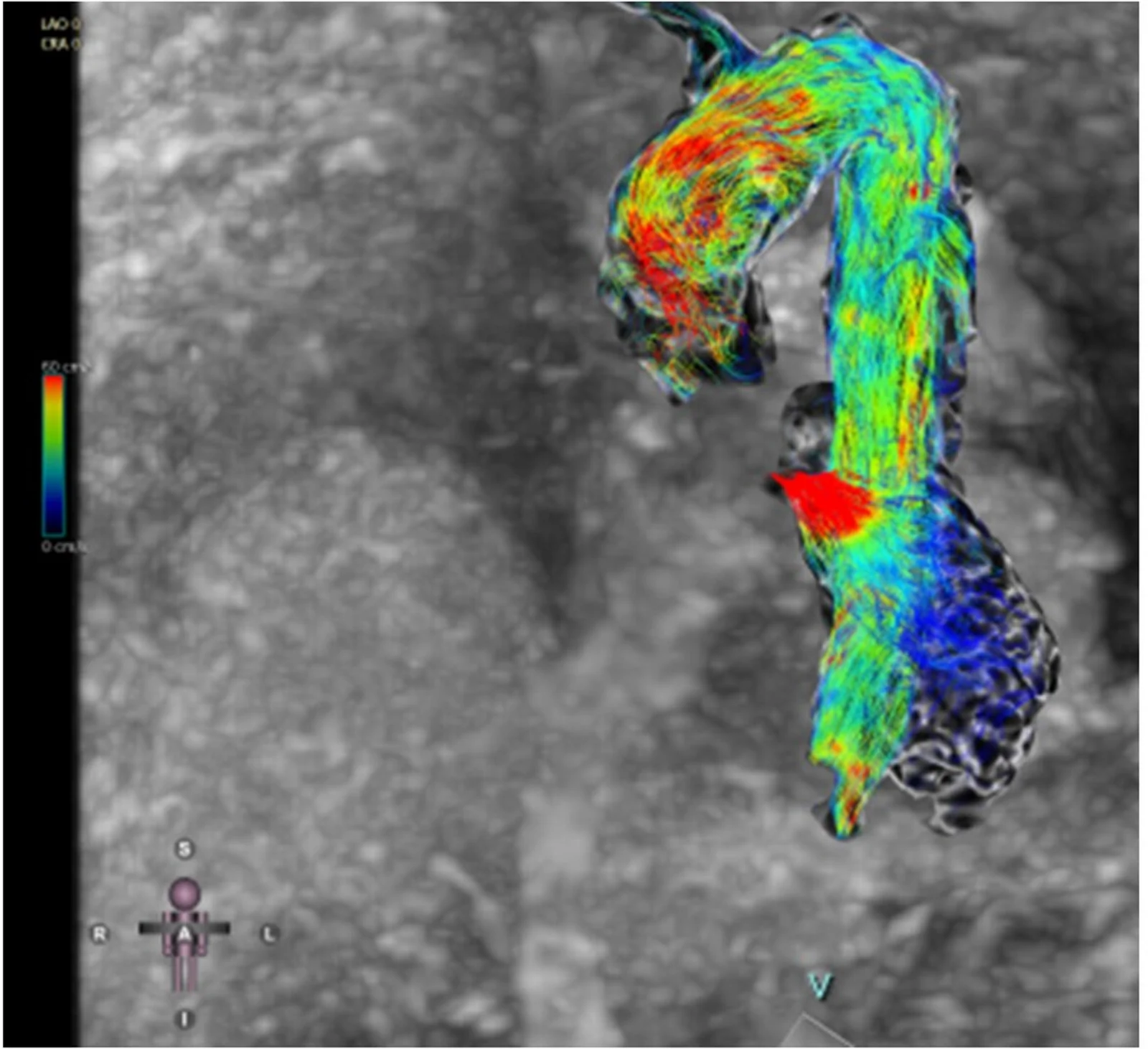
Four-dimensional flow magnetic resonance imaging. Four-dimensional flow magnetic resonance imaging (4D-flow MRI) streamline visualization showing accelerated systolic flow at the reconstructed left ventricular outflow tract (LVOT), corresponding to the narrowed segment identified on computed tomography, indicating functional LVOT stenosis

Postoperative contrast-enhanced computed tomography revealed significant narrowing of the LVOT at the level of the inverted graft, with a minimal diameter of 14 mm (Fig. 5A, B). Transthoracic echocardiography was performed postoperatively; however, adequate visualization of the LVOT was not achieved, and Doppler-derived assessment of flow velocity could not be reliably obtained. Therefore, four-dimensional (4D) flow magnetic resonance imaging (MRI) was performed as an adjunctive modality. 4D-flow MRI demonstrated accelerated systolic flow at the reconstructed LVOT and allowed quantitative assessment of flow velocity, indicating functional LVOT stenosis (Fig. 6). The postoperative course was uneventful, and the patient was transferred to a rehabilitation facility without signs of recurrent infection.

## Discussion

Redo aortic root surgery for infective endocarditis complicated by annular destruction remains technically challenging. Although extensive debridement is mandatory to eradicate infection, once annular continuity is lost, secure proximal reconstruction becomes difficult, and conventional techniques may fail [1–3].

In the present case, although the patient had a history of infective endocarditis with an annular abscess, intraoperative examination revealed no macroscopic evidence of active infection at the time of reoperation. The pseudoaneurysm cavity was surrounded by fibrotic tissue, and the aortic wall had been replaced by fragile but noninfected tissue. These findings suggest that the pseudoaneurysm and prosthetic valve dehiscence were the consequences of previous infection rather than ongoing inflammatory destruction. This distinction is important when interpreting postoperative anatomical and hemodynamic findings.

The graft insertion technique, originally described by Nakamura et al., was designed to achieve secure proximal fixation in cases with extensive annular damage [4]. Recent reports, including a midterm outcome study by Narita et al., have demonstrated favorable outcomes of this technique in redo aortic root surgery, with no recurrence of pseudoaneurysm or major aortic events [5]. Nevertheless, most previous studies have focused primarily on infection control and anastomotic security, with limited attention toward postoperative LVOT geometry.

**Fig. 7:**
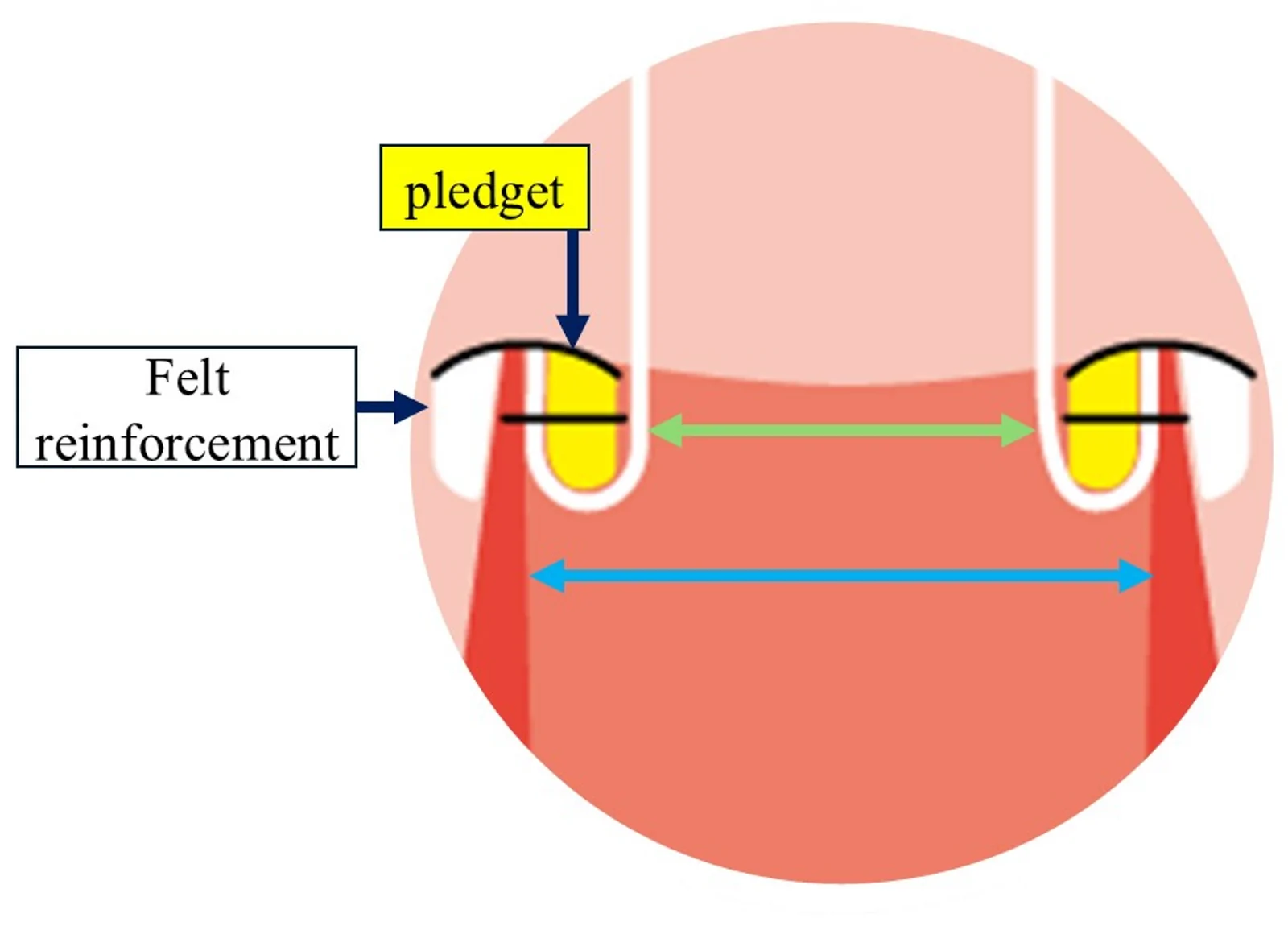
Schematic illustration of the mechanism of left ventricular outflow tract (LVOT) narrowing after the graft insertion technique. The blue arrow indicates the original LVOT diameter before reconstruction, whereas the green arrow indicates the reduced functional LVOT diameter after reconstruction. The yellow structures represent pledgets placed circumferentially at the proximal anastomosis, and the outer white layer represents felt reinforcement. After graft inversion and pull-through, these reinforcement materials occupy part of the luminal space and reduce the effective internal radius of the reconstructed LVOT

Furthermore, a ball sizer or Hegar dilator was not used during knot tying in this case. This maneuver is considered an important technical step to maintain luminal diameter, and its omission may have contributed to the development of LVOT stenosis. Importantly, the native LVOT was not markedly small prior to the initial surgery (minimum diameter, 21.7 mm; area, 5.18 cm²; Fig. 3), suggesting that the severe postoperative narrowing cannot be explained solely by baseline anatomy. Instead, these findings strongly indicate that the observed LVOT stenosis was primarily iatrogenic and related to the reconstruction technique. Several mechanisms may have contributed to LVOT narrowing in this case. First, the native LVOT geometry may have provided limited tolerance to additional narrowing. Second, the intraventricular portion of the inverted graft may have contributed to luminal reduction. However, the most relevant mechanism in this case was considered to be the circumferential placement of pledgeted mattress sutures and reinforcement materials at the proximal anastomosis. After graft inversion and pull-through, these materials are distributed circumferentially, effectively reducing the internal radius of the reconstructed LVOT. This mechanism is schematically illustrated in Fig. 7. Although a 24-mm vascular graft was used, the effective internal diameter of the reconstructed LVOT may be substantially smaller because of the cumulative thickness of the graft material, pledgets, and felt reinforcement. In our case, this resulted in a marked discrepancy between the expected and observed LVOT diameter.

This morphological finding was further supported by functional evaluation using 4D-flow MRI. Although echocardiographic assessment was attempted postoperatively, adequate visualization of the LVOT was not achieved, and Doppler-derived measurements could not be reliably obtained. In contrast, 4D-flow MRI enabled visualization of localized systolic flow acceleration and allowed quantitative assessment of flow velocity at the reconstructed LVOT. These findings confirm that the observed narrowing represented true functional LVOT stenosis rather than a transient or imaging-related artifact. Previous studies have also demonstrated the utility of 4D-flow MRI for investigating complex postoperative flow disturbances [6, 7].

Although the patient remained clinically stable, this case highlights an important pitfall of the graft insertion technique. Even when infection has been eradicated and an appropriately sized prosthetic valve is used, LVOT narrowing may occur as a consequence of the reconstruction itself. Therefore, surgeons should recognize that secure annular reconstruction may come at the cost of reduced LVOT area and should take precautions to minimize this risk.

## Conclusion

The graft insertion technique is a valuable option for redo aortic root reconstruction in cases with extensive annular destruction, even after infective endocarditis. Nonetheless, this case demonstrates that postoperative left ventricular outflow tract (LVOT) narrowing may occur as a consequence of the intraventricular portion of the graft and the proximal suture line. Therefore, meticulous technical attention is required to prevent this complication, including appropriate graft sizing and careful management of the proximal anastomosis. Postoperative multimodality imaging, particularly contrast-enhanced computed tomography and four-dimensional (4D) flow magnetic resonance imaging, may play an essential role in detecting functional LVOT stenosis and ensuring the hemodynamic safety of this technique.

## Data Availability

All data generated or analyzed during this study are included in this published article.

## Abbreviations

LVOT: Left ventricular outflow tract
GIT: Graft insertion technique
MRI: Magnetic resonance imaging
PVL: Paravalvular leakage
LCC: Left coronary cusp
RCC: Right coronary cusp
CABG: Coronary artery bypass grafting
